# An oscillating Min system in *Bacillus subtilis* influences asymmetrical septation during sporulation

**DOI:** 10.1099/mic.0.059295-0

**Published:** 2012-08

**Authors:** Ján Jamroškovič, Nad’a Pavlendová, Katarína Muchová, Anthony J. Wilkinson, Imrich Barák

**Affiliations:** 1 Institute of Molecular Biology, Slovak Academy of Sciences Dúbravská cesta 21, 845 51 Bratislava, Slovakia; 2 Structural Biology Laboratory, Department of Chemistry, University of York, York YO10 5YW, UK

## Abstract

The Min system plays an important role in ensuring that cell division occurs at mid-cell in rod-shaped bacteria. In *Escherichia coli,* pole-to-pole oscillation of the Min proteins specifically inhibits polar septation. This system also prevents polar division in *Bacillus subtilis* during vegetative growth; however, the Min proteins do not oscillate in this organism. The Min system of *B. subtilis* plays a distinct role during sporulation, a process of differentiation which begins with an asymmetrical cell division. Here, we show that oscillation of the *E. coli* Min proteins can be reproduced following their introduction into *B. subtilis* cells. Further, we present evidence that the oscillatory behaviour of the Min system inhibits sporulation. We propose that an alternative Min system mechanism avoiding oscillation is evolutionarily important because oscillation of the Min system is incompatible with efficient asymmetrical septum formation and sporulation.

## Introduction

Rod-shaped bacteria multiply by binary fission, in which the division septum forms with high precision at the cell’s centre. How the division machinery achieves such accuracy is a question of enduring interest. Assembly of FtsZ protomers into a circular structure, called the Z-ring, at the future division site is a prerequisite for cell division (Bi & Lutkenhaus, 1991). It is assumed that initiation of cell division is regulated at the step of FtsZ polymerization and Z-ring placement. Several FtsZ-interacting proteins modulate FtsZ polymerization, acting either to promote or to inhibit this process. MinC prevents FtsZ polymerization and acts as a direct block of polar division (de Boer *et al.*, 1989). In *min* mutant strains, polar cell division results in the formation of mixtures of ‘mini’ cell forms which lack chromosomes, and extended rods containing multiple nucleoids (Adler *et al.*, 1967; Reeve *et al.*, 1973).

The localization and activity of MinC are dependent on interactions with MinD, an ATPase that associates peripherally with the cytoplasmic membrane (de Boer *et al.*, 1991). MinC and MinD homologues are found in both the Gram-negative *Escherichia coli* and the Gram-positive *Bacillus subtilis*. MinD binds reversibly to negatively charged membrane lipids in an ATP-dependent manner (Hu *et al.*, 2002; Hu & Lutkenhaus, 2003; Barák *et al.*, 2008). It is unevenly distributed along the length of the cell, with the highest concentration of MinD and consequently also of MinC found at the cell poles (Marston *et al.*, 1998; Hu & Lutkenhaus, 1999; Raskin & de Boer, 1999a, b). In *E. coli*, this pattern of localization is determined by MinE. MinE tracks MinD and can be visualized as a ring-like structure at the periphery of the zone occupied by the MinCD complex at the cell pole (Fu *et al.*, 2001; Hale *et al.*, 2001). MinE binding to MinD is accompanied by displacement of MinC and stimulation of the ATPase activity (Hu & Lutkenhaus, 2001), leading to release of MinD from the membrane. Intracellularly, these events lead to net migration of MinD to the opposite cell pole, again followed by MinE, where the molecular events are repeated. This dynamic oscillation process, which takes place with a cycle time of 20–50 s, leads to a MinC concentration minimum at the cell’s centre, where cell division takes place (Hu and Lutkenhaus, 1999; Raskin & de Boer, 1999a, b; Fu *et al.*, 2001; Hale *et al.*, 2001; Juarez & Margolin, 2010; Di Ventura & Sourjik, 2011).

The Min system of *B. subtilis* features MinC (MinC_Bs_) and MinD (MinD_Bs_), but there is no MinE homologue. Instead, two proteins, MinJ and DivIVA, determine the polar localization of the MinCD complex (Edwards & Errington, 1997; Marston *et al.*, 1998; Bramkamp *et al.*, 2008; Patrick & Kearns, 2008). DivIVA recognizes and binds to negative membrane curvature generated at the newly forming cell poles during cell division, and it recruits the other Min system proteins so as to block the premature formation of a subsequent septum (Lenarcic *et al.*, 2009; Ramamurthi & Losick, 2009; Eswaramoorthy *et al.*, 2011). MinJ, a membrane protein, is recruited by DivIVA to the division site that will become the new cell pole, where it accumulates and serves as a localization signal for MinD (Bramkamp *et al.*, 2008; Patrick & Kearns, 2008).

DivIVA recruits a different set of proteins to the cell poles during sporulation, when it is required for proper segregation of the axial filament, a structure that is composed of elongated sister chromosomes anchored in the vicinity of their *ori* regions to opposite cell poles (Wu & Errington, 1994, 1998; Webb *et al.*, 1997). In this sporulation-specific chromosomal structure, RacA acts as a bridge between DivIVA at the cell pole and the *ori* region of the chromosome (Ben-Yehuda *et al.*, 2003; Wu & Errington, 2003). The implied switching of partners by DivIVA may serve to couple relief of inhibition of polar septum formation to faithful chromosome segregation during sporulation. Although deletion of *minD* has no observable effect on the efficiency of sporulation, the sporulation septum is often misplaced closer to mid-cell in MinD-deficient cells (Barák *et al.*, 1998; Thomaides *et al.*, 2001). At present, the details are not known of how the inhibitory effect of the Min system proteins on polar division is overcome during sporulation.

In *B. subtilis*, oscillation of the Min proteins has not been observed, indicating a different mechanism of cell division site recognition. Although the Min system in *B. subtilis* is not as conspicuously dynamic as that in *E. coli*, there is rapid binding and dissociation of MinD_Bs_ molecules at the membrane, and it is postulated that this is accompanied by MinD_Bs_ polymerization and depolymerization, respectively (Barák *et al.*, 2008). This characteristic of MinD_Bs_ is not so surprising given the high sequence identity between the MinD proteins of *B. subtilis* and *E. coli* and the observation of reversible ATP-dependent membrane binding by MinD_Ec_ (Drew *et al.*, 2005). The remaining *B. subtilis* Min system proteins are less dynamic, although rapid movement of MinC_Bs_ has been shown following formation of the cell division septum (Gregory *et al.*, 2008).

The different composition and mechanism of action of the Min systems in *E. coli* and *B. subtilis* raise interesting evolutionary questions concerning (i) why different mechanisms have evolved to achieve the common goal of disabling polar division, (ii) whether the two mechanisms evolved one from another and, if so, (iii) which of the Min systems appeared first. It is known that MinD_Ec_ partially complements MinD_Bs_, and that YFP–MinD_Ec_ expressed in *B. subtilis* localizes on helical trajectories in the same way as GFP–MinD_Bs_ (Barák *et al.*, 2008; Pavlendová *et al.*, 2010). This indicates that MinD_Ec_ is able to function together with the *B. subtilis* Min system, and more specifically, to bind to MinC_Bs_ (Pavlendová *et al.*, 2010). However, MinE is less promiscuous. It fails to form a ring-like structure or even to localize to the cell membrane of *B. subtilis*. Instead, the fluorescence signal from MinE–GFP is distributed throughout the cytoplasm, suggesting that the absence of MinE oscillation in *B. subtilis* is due to its failure to bind to MinD_Bs_.

Here, we show that in the presence of MinE we can reproduce the oscillation of MinD_Ec_ in *B. subtilis*. We also show that cells with oscillating MinD form spores inefficiently. This is not due to defects in signalling, as activation of the response regulator Spo0A occurs normally. Instead, it appears that the cells are affected at the stage of formation of the hallmark of sporulation – an asymmetrical septum. Sporulation would appear therefore to be incompatible with an oscillating Min system, and this may underpin the evolution of different mechanisms in the two bacterial types.

## Methods

### 

#### Bacterial strains, growth conditions and media.

Details of the construction of plasmids and descriptions of *B. subtilis* and *E. coli* strains used in this study are presented in Table 1 and Table S1 (available with the online version of this paper), respectively. Sequences of oligonucleotides used in this work are given in Table S2. Strains were grown in Luria broth (LB; Ausubel *et al.*, 1987) or Difco sporulation medium (DSM; Schaeffer *et al.*, 1965) at 37 °C or as stated in the text. DNA manipulations and transformations of *E. coli* were carried out by standard methods (Sambrook *et al.*, 1989). *B. subtilis* transformations were performed by the method of Harwood & Cutting (1990). The strains IB1230 and IB1242, with oscillating *E. coli* Min systems, tend to be unstable. These cells were always freshly prepared by transformation of chromosomal DNA from strain IB1228 into strains IB1111 and IB1112 (Table 1). When required, media were supplemented with the antibiotics ampicillin (100 µg ml^−1^), tetracycline (10 µg ml^−1^), kanamycin (10 µg ml^−1^ or 30 µg ml^−1^), spectinomycin (100 µg ml^−1^), chloramphenicol (5 µg ml^−1^), lincomycin (25 µg ml^−1^) or erythromycin (1 µg ml^−1^). Xylose at concentrations of 0.05–0.5 % (w/v) was used for induction of P_xyl_; for induction of expression from P_hyperspank_, 0.1–1 mM IPTG was used.

**Table 1.** Bacterial strains and their construction

Strain	Description	Reference or origin
***B. subtilis* strains**		
PY79	Prototrophic derivative of *B. subtilis* 168	Youngman *et al.* (1984)
MO649	*thrC* : : *cat*	Guérout-Fleury *et al.* (1996)
IB220	*spo0A* : : *kan*	Schmeisser *et al.* (2000)
IB1056	*minD_Bs_* : : *cat*	Barák *et al.* (2008)
IB1107	*minD_Bs_* : : *cat amyE* : : *P_xyl_–minE–gfp spc*	Pavlendová *et al.* (2010)
IB1110	*amyE* : : *P_hyperspank_–yfp–minD_Ec_ spc*	Pavlendová *et al.* (2010)
IB1111	*minD_Bs_* : : *cat amyE* : : *P_hyperspank_–yfp–minD_Ec_ spc*	Pavlendová *et al.* (2010)
IB1112	*minD_Bs_* : : *cat divIVA* : : *tet amyE* : : *P_hyperspank_–yfp–minD_Ec_ spc*	Pavlendová *et al.* (2010)
IB1155	*minD_Bs_* : : *cat amyE* : : *P_hyperspank_–yfp–minD_Ec_ spc thrC* : : *P_xyl_–minE–gfp erm*	IB1111 : : pSGminE
IB1244	*trpC2 minJ* : : *pMUTIN4*(*bla erm P_spac_lacZ lacI*) *minCD* : : *aph–A3 kan*	Bramkamp *et al.* (2008)
IB1228	*thrC* : : *P_xyl_–minE erm*	MO649 : : pNP–minE
IB1229	*amyE* : : *P_hyperspank_–yfp–minD_Ec_ spc thrC* : : *P_xyl_–minE erm*	IB1110 : : chr DNA IB1228
IB1230	*minD_Bs_* : : *cat amyE* : : *P_hyperspank_–yfp–minD_Ec_ spc thrC* : : *P_xyl_–minE erm*	IB1111 : : chr DNA IB1228
IB1242	*minD_Bs_* : : *cat divIVA* : : *tet amyE* : : *P_hyperspank_–yfp–minD_Ec_ spc thrC* : : *P_xyl_–minE erm*	IB1112 : : chr DNA IB1228
IB1362	*minJ* : : *kan*	PY79 : : pUS19–ΔminJ
IB1363	*minD_Bs_* : : *cat minJ* : : *kan amyE* : : *P_hyperspank_–minD_Ec_ spc thrC* : : *P_xyl_minE erm*	IB1230 : : chr DNA IB1362
IB1369	*minCD_Bs_* : : *kan amyE* : : *P_hyperspank_–yfp–minD_Ec_ spc*	IB1110 : : chr DNA IB1244
IB1370	*minCD_Bs_* : : *kan amyE* : : *P_hyperspank_–yfp–minD_Ec_ spc thrC* : : *P_Xyl_–minE erm*	IB1369 : : chr DNA IB1228
IB1371	*minCD_Bs_* : : *kan*	IB333 : : chr DNA IB1244
***E. coli* strains**		
MM294	*F^−^ endA1 hsdR17* (*rk^−^*, *mk*) *supE44 thi-1 recA^+^*	Meselson & Yuan (1968)
YLS1 : : pYLS68	D*minCDE P_lac_* : : *yfp–minD_Ec_* : : *minE–cfp*	Shih *et al.* (2002)
BTH101	*F^−^ cya-99 araD139 galE15 galK16 rpsL1*(*Str^r^*) *hsdR2 mcrA1 mcrB1*	Karimova *et al.* (1998)

#### Western blotting.

The intracellular levels of GFP, cyan fluorescent protein (CFP) and yellow fluorescent protein (YFP) fusion proteins were determined by Western blot analysis with an anti-GFP antibody (Roche Diagnostics) as described previously (Barák *et al.*, 2008). The expression of Spo0A was detected with polyclonal anti-Spo0A antibody. After reaching the stationary phase of growth, cells were collected and processed as described previously (Barák *et al.*, 2008).

#### Fluorescence microscopy.

Cells were grown to the desired phase and a small amount of culture was transferred to microscope slides covered with a thin layer of 1 % agarose in LB medium. When necessary, cells were concentrated by centrifugation (3 min×2.3 ***g***) and resuspended in a small volume of supernatant prior the examination. To visualize the cells and septal membranes, the cell cultures were stained with FM 4-64 dye (Molecular Probes) at a concentration of 1 µg ml^−1^. Fluorescence microscopy images were acquired using an Olympus BX61 microscope, equipped with an Olympus DP30BW camera and a spinning disc VivaTome Zeiss microscope. Olympus CellP imaging software and AxioVision 4.8.2.0 software were employed for image acquisition and analysis, and the Huygens Essential software package was used for image deconvolution.

#### Sporulation efficiency.

The sporulation efficiency was determined essentially as described in Harwood & Cutting (1990). Briefly, cultures were grown in DSM sporulation medium supplemented with 0.5 mM IPTG, 0.5 % xylose and half the dose of appropriate antibiotics at 37 °C for 24 h after inoculation. After heat treatment (85 °C, 15 min), cells were diluted in LB medium and plated onto LB agar plates. Colonies formed from outgrowing spores on these plates represent cells that were able to sporulate and thus survive the heat treatment. These experiments were repeated at least three times. The sporulation efficiency was defined in terms of c.f.u. as follows: (c.f.u. of spores/viable c.f.u.)/(wild-type viable spores/wild-type total viable c.f.u.) and compared with the sporulation efficiency of the wild-type strain, which was taken as 100 %.

#### Bacterial two-hybrid system.

Fusions of *E. coli* MinC, MinD and MinE proteins to the T25 and T18 fragments of adenylate cyclase were constructed in the bacterial adenylate cyclase-based two-hybrid (BACTH) system (Karimova *et al.*, 1998). To amplify genes of interest, the primer pairs minCecB2HS and minCecB2HE, minDecB2HS and minDecB2HE or minEecB2HS and minEecB2HE were used with chromosomal DNA from *E. coli* MM294 strain as template (Meselson & Yuan, 1968). Amplified genes were cloned into the *Eco*RI and *Bam*HI sites of plasmids pKT25 or pKNT25 and pUT18C or pUT18. Plasmids with T25 and T18 fusions to *B. subtilis minC*, *minD*, *minJ* and *divIVA* were a kind gift from Dr Richard Daniel, Newcastle University, UK. To test for protein–protein interactions, transformants of *E. coli* BTH101 (adenylate cyclase-deficient strain) were plated onto LB plates supplemented with 40 µg X-Gal ml^−1^, 0.1 mM IPTG, 100 µg ampicillin ml^−1^ and 30 µg kanamycin ml^−1^, and grown for 24–72 h at 30 °C. To detect interactions, the BACTH system protocol was followed.

#### Quantitative β-galactosidase assay.

β-Galactosidase activity was measured as described by Miller (1972) with an extra wash step added. To eliminate error due to the effects of different carbon sources in the growth medium, the cells were pelleted and resuspended in an assay buffer prior to further processing.

## Results

### *E. coli* MinD oscillation in *B. subtilis*

**Fig. 1.**
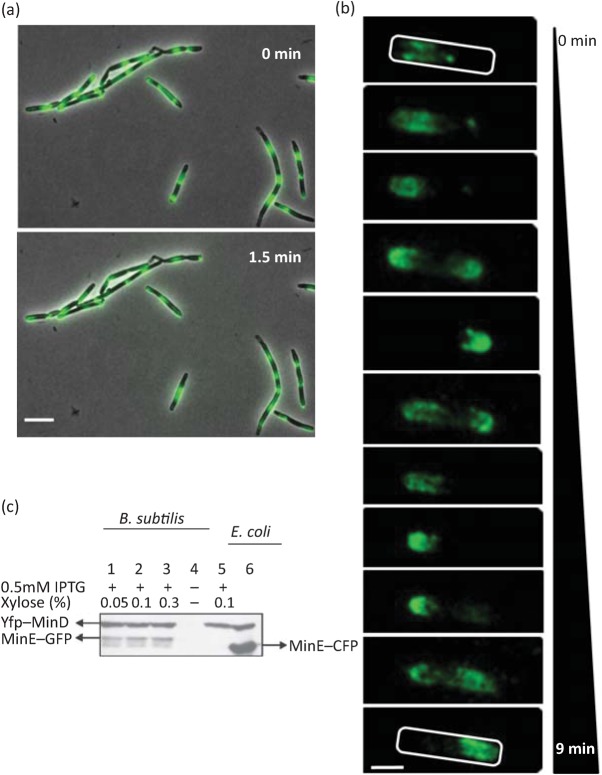
*E. coli* MinD can oscillate in the presence of MinE in *B. subtilis.* (a) Fluorescence micrographs showing localization of YFP–MinD_Ec_ in *B. subtilis* strain IB1242 (Δ*minD_Bs_* Δ*divIVA yfp–minD_Ec_ minE*). In most cells, oscillation of YFP fluorescence could be observed, although in some cells the fluorescence signal appears in the form of dots with reduced mobility. The images were taken with an Olympus BX61 microscope. Two pictures were taken 1.5 min apart. Scale bar, 5 µm. (b) Localization of YFP–MinD_Ec_ in a single cell of strain IB1230 (Δ*minD_Bs_ yfp–minD_Ec_ minE*). Images were captured using an Olympus BX61 microscope over a period of 9 min and the frames were deconvolved using Huygens Essential software. Scale bar, 1 µm. (c) Relative quantification of YFP–MinD (upper band) and MinE–GFP (lower band, lanes 1–3) in *B. subtilis* and MinE–CFP (lower band, lane 6) in *E. coli* by Western blotting. Anti-GFP antibody was used for detection of YFP–MinD, MinE–GFP and MinE–CFP. Lanes 1–3 represent *B. subtilis* strain IB1155 (Δ*minD_Bs_ yfp–minD_Ec_ minE–gfp*) in which expression of *yfp–minD* is induced with 0.5 mM IPTG and *minE–gfp* is induced with three different concentrations of xylose, ranging from 0.05 to 0.3 %. Lane 4 represents a negative control, strain IB1056 (Δ*minD_Bs_*). Lane 5 is strain IB1230 (Δ*minD_Bs_ yfp–minD_Ec_ minE*) with expression induced using 0.5 mM IPTG and 0.1 % xylose. Lane 6 represents *E. coli* strain YLS1 : : pYLS68 grown as described elsewhere (Shih *et al.*, 2002).

Through a series of genetic manipulations and adjustments to growth conditions, detailed below, we have been able to generate Min system oscillation in *B. subtilis*. This phenomenon is observed in the majority, if not all, of the cells in the population and occurs with an oscillation cycle time similar to that observed in *E. coli* (Fig. 1a, Movie S1).

MinD_Ec_ does not oscillate in the absence of MinE in *E. coli* (Hu & Lutkenhaus, 2001), nor does it do so when introduced into *B. subtilis* (Pavlendová *et al.*, 2010). We therefore examined the effect of introducing MinD_Ec_ together with MinE into *B. subtilis* by constructing strains expressing *yfp–minD_Ec_* and *minE* in a wild-type (IB1229) and a *minD_Bs_* deletion (IB1230) background. In many cells, we observed YFP–MinD_Ec_ foci close to the cell membrane, especially in strain IB1229. Movement of these ‘dots’ was generally confined to a small local region (Movie S2), and occasionally the dots relocalized towards one of the cell poles. In IB1230 cells, YFP–MinD_Ec_ movement reminiscent of oscillation in *E. coli* was visible, especially in shorter cells (Fig. 1b, Movie S3). Since overexpression of Min proteins causes cell elongation (Marston & Errington, 1999; Pavlendová *et al.*, 2010), the longer cells exhibiting the brightly fluorescing dots are likely to have higher YFP–MinD_Ec_ concentrations.

Higher concentrations of MinD_Ec_ and MinE may interfere with the function of the Min system by biasing the proportions of the complexes formed. In addition, interaction among *E. coli* and *B. subtilis* Min system components may cause slower movement of YFP–MinD_Ec_. In *E. coli*, the period of the Min oscillation cycle is 20–50 s (Raskin & de Boer, 1999a; Touhami *et al.*, 2006). To compare the oscillation times in *E. coli* and *B. subtilis,* we timed the YFP–MinD_Ec_ oscillation cycle in *E. coli* strain Δ*minCDE P_lac_* : : *yfp–minD_Ec_* : : *minE–cfp* (YLS1 : : pYLS68) (Shih *et al.*, 2002). In our hands, oscillation was observed with a period of about 1 min at room temperature. In contrast, the oscillation of YFP–MinD_Ec_ in *B. subtilis* Δ*minD_Bs_ yfp–minD_Ec_ minE* (IB1230) cells was slower at 1.5–3.5 min per cycle. Increasing the temperature to 30 °C, a change that in *E. coli* results in faster oscillation (from a cycle time of 20 s at 22 °C to 8 s at 30 °C; Touhami *et al.*, 2006), did not significantly enhance the oscillation frequency of YFP–MinD_Ec_ in *B. subtilis*. We reasoned that the presence of *B. subtilis* DivIVA or MinJ might be limiting the mobility of YFP–MinD_Ec_. To test this idea, we produced YFP–MinD_Ec_ and MinE in a *B. subtilis* strain in which either *minD* and *divIVA* (Δ*minD_Bs_* Δ*divIVA yfp–minD_Ec_ minE,* IB1242) or *minD* and *minJ* (Δ*minD_Bs_* Δ*minJ yfp–minD_Ec_ minE*, IB1363) were deleted. In these cells, the period of the oscillation cycle was essentially unchanged (1.5–3 min), but oscillation was observed in almost all cells (Fig. 1a, Movie S1).

Next we explored the possibility that the lower frequency of YFP–MinD_Ec_ oscillation in the *B. subtilis* system was caused by perturbations in the concentration ratios of the Min proteins. In the *B. subtilis* strains described here, YFP–MinD_Ec_ and MinE were expressed from the P_hyperspank_ and P_xyl_ promoters, respectively, while in *E. coli* YLS1 : : pYLS68, both genes were transcribed from the P_lac_ promoter. To compare MinDE expression levels in *B. subtilis* and in *E. coli*, we performed Western blot analysis. It is possible to visualize both MinD_Ec_ and MinE on one blot using a monoclonal anti-GFP antibody, in a strain where both MinD_Ec_ and MinE are in fusion with fluorescent proteins (Δ*minD_Bs_ yfp–minD_Ec_ minE–gfp*, IB1155). Under induction conditions similar to those used for the microscopy experiments (0.5 mM IPTG and 0.1 % xylose), it can be seen in Fig. 1(c) that while the concentrations of YFP–MinD_Ec_ (upper bands in lanes 1, 2, 3, 5 and 6) are similar in both systems, the concentration of MinE–CFP (lower band, lane 6) in *E. coli* strain Δ*minCDE P_lac_* : : *yfp-minD_Ec_* : : *minE–cfp* (YLS1 : : pYLS68) is higher than the concentration of MinE–GFP in *B. subtilis* strain expressing both YFP–MinD_Ec_ and MinE–GFP (IB1155) (lower band, lanes 1, 2 and 3). Although significant differences in the MinE–GFP expression levels under the three induction conditions tested (Fig. 1c, lanes 1, 2 and 3) were not observed, induction with 0.1 % xylose led to the highest YFP–MinD_Ec_ oscillation frequency, which approached one oscillation period per minute in many cells of Δ*minD_Bs_* (IB1230) and Δ*minD_Bs_* Δ*divIVA* (IB1242) *B. subtilis* strains. These experiments show that in the presence of MinE, YFP–MinD_Ec_ oscillates in *B. subtilis* and that the characteristics of the oscillation process closely reproduce the oscillation behaviour of the Min system observed in *E. coli.*

### Dynamic MinD inhibits sporulation

Over several days on DSM agar plates, colonies formed by strain Δ*minD_Bs_ yfp–minD_Ec_ minE* (IB1230) remained brighter coloured than those formed by wild-type *B. subtilis* cells, which became darker coloured as the cells sporulated. This suggested that IB1230 cells were impaired in sporulation. We therefore measured the sporulation efficiency of *B. subtilis* cells expressing the *E. coli* Min proteins. Interestingly, the sporulation efficiency of strain IB1230 is 10-fold lower (9 %) than that of wild-type cells (Table 2), suggesting that pole-to-pole oscillation of MinD inhibits spore formation. The sporulation efficiency of the strain Δ*minD_Bs_* Δ*divIVA yfp–minD_Ec_ minE* (IB1242), which gives the highest YFP–MinD_Ec_ oscillation frequency, was not tested, as *divIVA* mutants are already impaired in sporulation (Thomaides *et al.*, 2001).

**Table 2.** Sporulation efficiency of *B. subtilis* strains

Strain	Sporulation efficiency	Oscillation	*minD_Bs_*	*minC_Bs_*	*minD_Ec_*	*minE*
PY79	100 %	−	+	+	−	−
IB1056	85±1.9 %	−	−	+	−	−
IB1371	88.8±0.9 %	−	−	−	−	−
IB1111	85.4±1.9 %	−	−	+	+	−
IB1107	56.0±12.0 %	−	−	+	−	+
IB1229	53.4±17.5 %	+/−	+	+	+	+
IB1230	8.8±2.5 %	+	−	+	+	+
IB1370	1.7±0.9 %	+	−	−	+	+

Next, we inspected cells that retained wild-type *minD_Bs_* (*yfp–minD_Ec_ minE*, IB1229). The sporulation efficiency of these cells was lower (53 %) than that of the wild-type but significantly higher than that of the Δ*minD_Bs_ yfp–minD_Ec_ minE* (IB1230) strain (Table 2). It is possible that higher levels of MinD (MinD_Bs_ plus MinD_Ec_) lead to less efficient oscillation and thus to higher sporulation efficiency than that of strain IB1230. To test this possibility, we reinvestigated the sporulation efficiency of strain Δ*minD_Bs_ yfp–minD_Ec_ minE* (IB1230) under conditions in which the expression of YFP–MinD_Ec_ was increased by addition of 1 mM IPTG. Increased expression of MinD_Ec_ had no effect on the sporulation efficiency, which remained at 9 %. It seems therefore that it is the presence of MinD_Bs_ per se that causes the partial rescue of sporulation in strain *yfp–minD_Ec_ minE* (IB1229). When we examined the localization of YFP–MinD_Ec_ in this strain, we found that in most cells the YFP fluorescence appeared in the form of spots close to the membrane. In comparison with strain Δ*minD_Bs_ yfp–minD_Ec_ minE* (IB1230), clear YFP–MinD_Ec_ oscillation was visible in fewer cells. This observation implies that MinD_Bs_ binds to MinD_Ec_ and inhibits its MinE-induced oscillation.

**Fig. 2.**
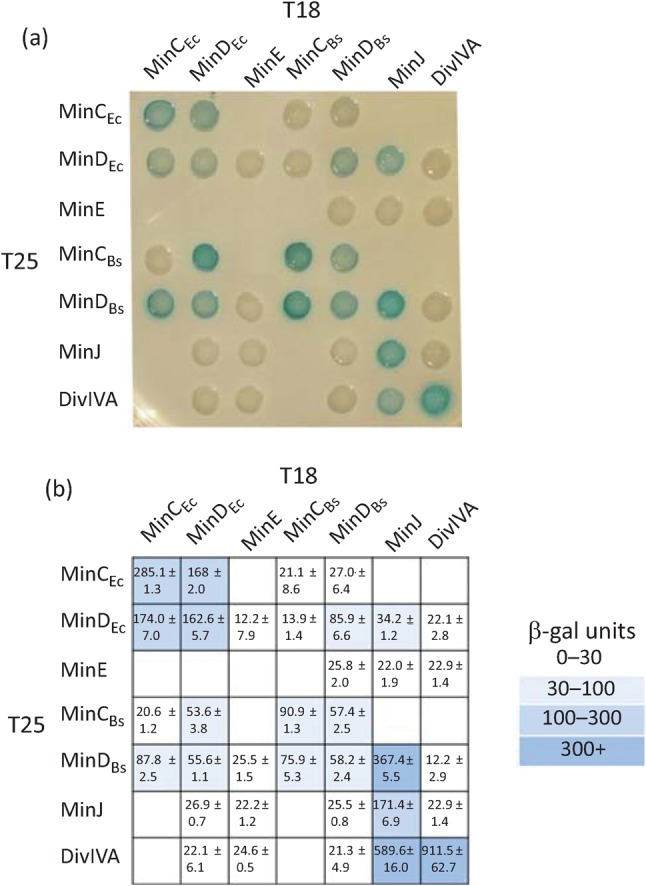
Interactions of Min proteins from *E. coli* and *B. subtilis. E. coli* strain BTH101 (Δ*cya*) was co-transformed with plasmids containing the indicated fusions of *E. coli* and *B. subtilis min* genes and *divIVA* to adenylate cyclase fragments T18 and T25. (a) Colonies spotted onto selective X-Gal plates indicate positive (blue) and negative (white) interactions, respectively. (b) The strength of each interaction was quantified by β-galactosidase assay. Numbers indicate Miller units of activity and represent the mean±sd of activity from at least three measurements. Positive interactions are marked by a range of blue colours, as indicated in the key.

We confirmed the implied interaction between the *E. coli* and *B. subtilis* MinD proteins using the bacterial two-hybrid system (Fig. 2). Overall, it seems that oscillation of MinD_Ec_ correlates with the lower sporulation frequency. Supporting this assertion, in a control Δ*minD_Bs_* strain expressing MinD_Ec_ in the absence of MinE (Δ*minD_Bs_ yfp–minD_Ec_*, IB1111), where MinD_Ec_ does not oscillate, sporulation is unimpaired (Table 2). This result excludes the possibility that the mere presence of MinD_Ec_ inhibits sporulation. In a control strain deleted for MinD_Bs_ (Δ*minD_Bs_*, IB1056), the sporulation efficiency is only slightly decreased. In a strain expressing MinE alone (Δ*minD_Bs_ minE–gfp*, IB1107), the sporulation efficiency decreased to 56 %.

The simplest explanation for the decreased sporulation efficiency of strain IB1230 (Δ*minD_Bs_ yfp–minD_Ec_ minE*) is that interactions with MinD_Ec_ (Fig. 2) induce oscillation of MinC_Bs_, leading to increased MinC concentrations at the cell poles which prevent asymmetrical septation. However, the sporulation efficiency of strain IB1370 (Δ*minC_Bs_* Δ*minD_Bs_ yfp–minD_Ec_ minE*), in which both MinD_Bs_ and MinC_Bs_ are deleted, is even lower (2 %) than that observed in strain IB1230 (Δ*minD_Bs_ yfp–minD_Ec_ minE*). Thus, MinC oscillation does not explain the observed lowering of the sporulation efficiency of strain Δ*minD_Bs_ yfp–minD_Ec_ minE* (IB1230), in which MinD oscillation takes place. It is important to note that in MinD-deficient *B. subtilis* cells, the sporulation septum is often misplaced closer to mid-cell (Barák *et al.*, 1998; Thomaides *et al.*, 2001). In addition, MinCD depletion causes loss of polarity in SpoIIIE-mediated chromosome translocation (Sharp & Pogliano, 2002). However, neither of these two phenotypes is associated with as obvious a reduction in the sporulation efficiency as that observed in strains IB1230 or IB1370 (Table 2). Thus, we assume that the heterologous, oscillating Min system has an additional inhibitory effect on the complex process of sporulation either during asymmetrical septum formation or in the later stages.

### Oscillating Min proteins block sporulation by inhibition of polar septum formation

**Fig. 3.**
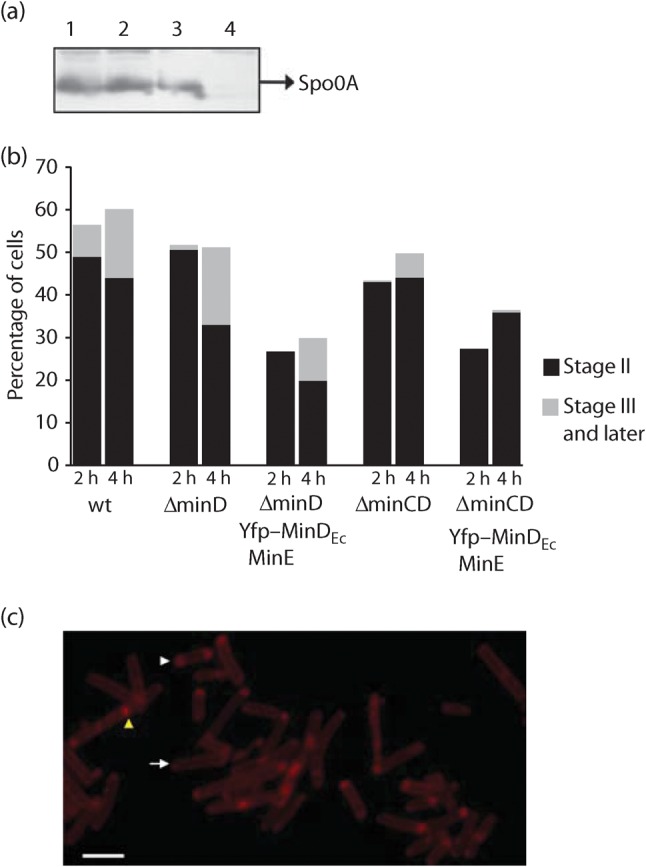
Sporulation block is caused by inefficient asymmetrical septum formation. (a) Western blot with anti-Spo0A antibody, illustrating that the levels of Spo0A in strains where oscillation was observed (IB1242, lane 1; IB1230, lane 2) are similar to levels observed in wild-type *B. subtilis* strain PY79 (lane 3). This indicates that the block in sporulation is not at the stage of sporulation initiation. No Spo0A was detected in the control strain Δ*spo0A* IB220 (lane 4). (b) To inspect the cells for asymmetrical septum formation, cells were harvested at hour 2 and hour 4 of sporulation, and the membranes were stained using FM4-64 dye. The cells were classified into three groups. First, cells with asymmetrical septa, representing stage II of sporulation (black); cells with a clear minicell phenotype were excluded. Second, cells in later stages of sporulation, stages III and later (grey). The rest of the cells, representing vegetative cells, are not marked. Cells of *B. subtilis* IB1230 and IB1370 are visibly blocked or delayed in the formation of polar septa. (c) Example of FM4-64-stained cells of IB1370 at hour 2 of sporulation. The arrow indicates a vegetative cell, the white triangle shows a cell in stage II and the yellow triangle a cell in stage III of sporulation. Bar, 3 µm.

The master regulator of sporulation initiation is Spo0A, a response regulator that is phosphorylated by a multi-component phosphorelay (Hoch, 1993; Perego & Hoch, 2002). Phosphorylated Spo0A binds to specific promoter regions (‘0A boxes’), and activates or represses the expression of scores of genes required for sporulation (reviewed by Piggot & Losick, 2002; Barák *et al.*, 2005). To test whether strains exhibiting oscillation of MinD_Ec_ are defective in sporulation initiation, we examined Spo0A expression levels by Western blotting (Fig. 3a). Spo0A is present at similar levels in strain Δ*minD_Bs_ yfp–minD_Ec_ minE* (IB1230), which exhibits Min system oscillation, and in the wild-type strain (PY79), which does not. Indeed in all strains examined, Spo0A was detected at normal levels, the exception being the control strain in which *spo0A* has been deleted (IB220; Schmeisser *et al.*, 2000). Thus the reduced sporulation efficiency associated with the oscillating Min system is not caused by perturbations in the level of Spo0A. Since *spo0A* expression is positively autoregulated (Molle *et al.*, 2003), normal Spo0A levels indicate that the activity of Spo0A, and the system of proteins that activate it, is unaffected by the oscillating Min system.

A more likely explanation for the lowered sporulation efficiency is a defect in polar cell division. Oscillating MinD_Ec_ is expected to bind to MinC_Bs_, thus conferring pole-to-pole oscillation on the cell division inhibitor, which would prevent polar septum formation. This hypothesis was tested by membrane staining. Cells of the wild-type strain (PY79) and strain Δ*minD_Bs_ yfp–minD_Ec_ minE* (IB1230) were grown until hours 2 and 4 of sporulation, and the membranes were stained with the dye FM4-64. The pattern of staining defined three discernible cell classes: (i) cells with a polar septum (stage II), (ii) cells in the later stages of sporulation (stage III and later), and (iii) vegetative cells.

For the wild-type strain, after 2 h, 44 % of the cells had not entered into sporulation, 49 % of cells showed a clear polar septum and the remaining cells were in stage III or later (Fig. 3b). Cells of strains Δ*minD_Bs_ yfp–minD_Ec_ minE* (IB1230) and Δ*minC_Bs_* Δ*minD_Bs_ yfp–minD_Ec_ minE* (IB1370), which harbour the oscillating *E. coli* Min system components, were noticeably impaired in the formation of asymmetrical septa. In the second hour of sporulation, forespores in stage III or later were not observed, and an asymmetrical septum was observed in only about 27 % of the cells (IB1230 and IB1370). As mentioned previously, the sporulation efficiency of strain Δ*minD_Bs_ yfp–minD_Ec_ minE* (IB1230) is around 9 %. This indicates that even though polar septa are forming in 27 % of these cells at hour 2 of sporulation, only one-third of these give rise to resistant spores. In summary, *B. subtilis* cells, in which the *E. coli* Min system proteins oscillate, initiate sporulation normally but are impaired in sporulation septum formation.

## Discussion

Regulation of cell division site placement is an intensively studied phenomenon in the model organisms *E. coli* and *B. subtilis*. The Min system serves in both classes of organisms as an efficient blockade of unwanted polar septation, but quite different mechanisms of Min system action are postulated. In *E. coli*, pole-to-pole oscillation of MinCDE creates a concentration gradient of the cell division inhibitor MinC, with the highest concentration at the cell poles, where septation is restricted (Marston *et al.*, 1998; Hu & Lutkenhaus, 1999; Raskin & de Boer, 1999a, b; Hale *et al.*, 2001). In contrast, the MinCDJ–DivIVA complex localizes at the newly formed cell poles and persists at the polar positions in *B. subtilis* (Edwards & Errington, 1997; Marston *et al.*, 1998; Bramkamp *et al.*, 2008; Patrick & Kearns, 2008, Eswaramoorthy *et al.*, 2011).

The dynamics of MinD localization and reversible membrane binding are integral to the function of both Min systems. The determinant of MinD affinity for the membrane is an amphipathic α-helix at its C terminus (Hu & Lutkenhaus, 2003; Szeto *et al.*, 2003). MinD_Bs_ preferentially binds to membranes enriched in negatively charged lipids, such as phosphatidylglycerol, which are helically arranged (Barák *et al.*, 2008). MinD_Ec_ also oscillates on a helical trajectory, although it is not known whether helical phosphatidylglycerol domains exist in the cytoplasmic membrane of *E. coli* (Shih *et al.*, 2003). The phospholipid composition of the membranes of *E. coli* and *B. subtilis* is strikingly different. Phosphatidylglycerol represents 40 and 20 % and cardiolipin 24 and 4 % of the membrane phospholipids in *B. subtilis* and *E. coli*, respectively (Kusters *et al.*, 1991; López *et al.*, 1998).

These comparisons raise many interesting questions, including whether the *E. coli* Min system would oscillate following its transplantation into *B. subtilis*. Elsewhere, oscillation of MinD from Gram-negative *Neisseria gonorrhoeae* was observed in *E. coli* (Ramirez-Arcos *et al.*, 2002). Oscillation is an intrinsic property of the Min proteins of *E. coli*, as shown by the elegant studies on flat membrane systems (Loose *et al.*, 2008). Here we have shown that the *E. coli* Min system behaves dynamically in Gram-positive *B. subtilis*. We discovered conditions under which *E. coli* MinDE oscillation in *B. subtilis* closely resembles oscillation in *E. coli*. Oscillation of the Min system proteins is therefore not restricted by the different membrane compositions of *E. coli* and *B. subtilis*. This prompts the subsidiary question of why separate mechanisms have evolved to achieve the same goal. One reason could be the incompatibility of Min system oscillation with sporulation. We observed a significant decrease in the sporulation efficiency of *B. subtilis* cells in which oscillation of *E. coli* MinD was observed. The defect is not manifested at the stage of sporulation initiation, since expression and activation of the master regulator of sporulation, Spo0A, are unaffected. In contrast, the capacity of the cells to form intact polar septa was impaired, and this was also observed in a strain in which both MinD_Bs_ and MinC_Bs_ were depleted. Taken together, these results demonstrate that expression of heterologous, oscillating Min proteins restricts polar septum formation by a mechanism that is MinC-independent.

For sporulation to occur there has to be a mechanism for liberating the polar septation sites from the division-inhibitory activity of the Min system. A key factor at this stage is DivIVA, with its alternative functions in vegetative cell division and in sporulation. We speculate that upon binding to RacA, DivIVA loses its capacity to bind to the Min proteins and confine them to the cell poles. This delocalization of the Min proteins would then allow SpoIIE-dependent assembly of FtsZ-rings (Z-rings) at the site of asymmetrical septation. The presence of the oscillating Min system, transplanted from *E. coli*, has a negative effect on either asymmetrical septum formation or the later stages of the sporulation process, or on both.

### Evolution of Min systems and sporulation

The evolutionary implications of these observations are that bacteria which form endospores will have DivIVA/MinJ rather than MinE as the auxiliary component(s) of MinCD. Until recently, sporulation was thought to be restricted to species of Gram-positive bacteria. As shown in Table S3, the genomes of all Gram-positive endospore-forming bacteria encode a DivIVA homologue and most also encode a MinJ homologue. Interestingly, most of the sporulating *Clostrideae* sp. also possess a MinE homologue (Table S3). However, it is not known whether these MinE proteins are functional, if they are part of Min systems which oscillate, and what, if any, interplay there is with the DivIVA/MinJ system during vegetative growth and sporulation.

The chromosomes of almost all rod-shaped Gram-negative bacteria encode a MinE homologue, and some encode homologues of DivIVA (Rothfield *et al.*, 2005; Table S3). Gram-negative bacteria have hitherto been considered to be non-sporulating, with the possible exception of a sparsely documented example in *Thermus*. In addition, *Myxococcus* forms spores by converting the rod-shaped vegetative cell into a spherical spore without prior asymmetrical division (Kaiser, 2003).

From the available data it is hard to deduce which Min system evolved from which, just as we do not know whether the common ancestor of Gram-positive and Gram-negative bacteria possessed these different characteristics. We can speculate that the Min systems either evolved separately or, more likely, evolved together in Gram-positive bacteria for the alternate life cycles of vegetative growth and sporulation, as MinE and DivIVA/MinJ are present in most *Clostrideae* sp. If this assumption is true, then most probably Gram-negative bacteria evolved from a Gram-positive bacterium. This notion is supported by the recent fascinating description of the cell membrane structures of *Acetonoma longum* (evolutionarily a close relative of *Clostrideae* sp.) during sporulation and spore outgrowth (Tocheva *et al.*, 2011). Those authors show that during sporulation the inner membrane of the mother cell is inverted and transformed to become an outer membrane of the germinating cell. Their results point to sporulation as a mechanism by which the bacterial outer membrane could have arisen. If *A. longum* is the missing link between single- and double-membraned bacteria, it is not surprising that it possesses the two cell-division regulatory systems that characterize Gram-positive and Gram-negative micro-organisms. Further work is needed to address whether and how these two systems function together in the same cell.
